# Design, synthesis and antitubercular evaluation of novel 5,6-diphenyl-1,2,4-triazine-piperazine derivatives targeting mycobacterial dihydrofolate reductase

**DOI:** 10.5599/admet.3322

**Published:** 2026-06-08

**Authors:** Uday Thakkar, Moksh Shah, Pratik Khona, Harnisha Patel, Mange Ram Yadav, Salman Patel, Chanchal Singh, Renuka Bhamre, Afzal Nagani

**Affiliations:** 1 Parul Institute of Pharmacy, Parul University, Vadodara, Gujarat, India; 2 Faculty of Pharmacy, Parul University, Vadodara, Gujarat, India; 3 Krishna School of Pharmacy, KPGU, Vadodara, Gujarat, India; 4 Research and Development Chemist, Purical Inc., Etobicoke, Canada; 5 Research and Development Cell, Parul University, Vadodara, Gujarat, India

**Keywords:** Antitubercular, density function theory, Mtb-DHFR inhibitor, molecular docking, molecular dynamic simulation

## Abstract

**Background and purpose:**

Tuberculosis (TB), a serious global health concern, continues to contribute to the global health burden, underscoring the urgent need to discover potent antitubercular agents. In the present study, a series of novel 5,6-diphenyl-1,2,4-triazine-piperazine derivatives were synthesized, and their potential for anti-tubercular activity was assessed.

**Experimental approach:**

The antitubercular potential of the synthesized compounds was assessed using the microplate alamar blue assay (MABA). Cytotoxicity studies were carried out on RAW 264.7 macrophages. To assess the mode of action of the synthesized compounds, docking analysis of the active compounds was performed on two key enzymes of *Mycobacterium*, decaprenylphosphoryl-β-D-ribose 2′-epimerase and *Mycobacterium tuberculosis*-dihydrofolate reductase (*Mtb*-DHFR). Furthermore, molecular dynamics simulations and density functional theory calculations were performed to validate the stability of the ligand–protein complexes and the electronic properties of the lead compounds.

**Key results:**

The results revealed promising anti-tubercular activity for the synthesized compounds. Among these, compounds 2-((5,6-Diphenyl-1,2,4-triazin-3-yl)thio)--1-(4-(4-fluorobenzoyl)piperazin-1-yl)ethan-1-one (FP3) and 2-((5,6-diphenyl-1,2,4-triazin-3-yl)thio)-1-(4-(4-methylbenzoyl) piperazin--1-yl)ethan-1-one (FP8) exhibited potent activity with an minimum inhibitory concentration of 1.6 μg mL^-1^, along with promising cytotoxicity profiles against RAW 264.7 macrophage cells. Docking studies revealed potent docking scores compared to the standard, thereby confirming the involvement of the DHFR enzyme. Interaction analysis revealed stable hydrogen-bond interactions, π-π stacking, and hydrophobic interactions with the active-site residues of the DHFR enzyme. Additionally, molecular dynamics simulation validated the stability of the interactions and the structural integrity of the complexes, while density functional theory calculations supported the desirable electronic properties of the lead compounds.

**Conclusion:**

The combined results of the experiments and calculations validated the potential of FP3 and FP8 as DHFR-targeted antitubercular agents. The study highlights the potential of 5,6-diphenyl-1,2,4-triazine-piperazine derivatives as promising candidates for further anti-tubercular drug development.

## Introduction

Tuberculosis (TB), being a major health concern at the global level, has seen a rise in the number of TB infections, deaths, and drug-resistant strains of TB during the period from 2020 to 2022 due to the COVID-19 pandemic [1,2]. The appearance of drug-resistant strains of TB has increased the urgency of the search for new drugs that can increase the number of existing drug options against TB. Existing TB drugs are effective against only a limited number of non-replicating bacterial populations because they target processes essential for the growth and replication of *Mycobacterium tuberculosis*. Therefore, the duration of treatment regimens of drug-susceptible TB (DS-TB) and drug-resistant TB (DR-TB) is long, taking months to cure the disease. In addition, the existing regimens of DR-TB are associated with low cure rates and high toxicity, making it imperative to look for effective and safe alternatives against DR-TB [3,4]. *Mycobacterium tuberculosis* (*Mtb*), *Mycobacterium caprae*, *Mycobacterium canettii*, *Mycobacterium bovis, Mycobacterium pinnipedii, Mycobacterium africanum*, and *Mycobacterium microti* are among the *Mycobacterium* species that cause tuberculosis. *Mtb* is the most prevalent of all *Mycobacterium* species and is responsible for causing pulmonary infections, but *Mtb* can also infect other vital organs [5-7].

Despite the identification of a number of natural and synthetic compounds with potent activity against resistant strains of *Mtb*, many of them have failed in early clinical trials and thus far, there are only a handful of drugs that have been registered for the therapy of TB during the last 50 years. The long duration of therapy and the associated toxic effects of existing antimycobacterial agents highlight the urgent need for the discovery of newer, safer chemotherapeutic agents [8,9].

Significant improvements have been made in understanding the molecular and cellular biology of TB bacteria. This has led to the identification of several molecular targets involved in the development and progression of TB. This has also led to the development of novel, rationally designed molecularly targeted agents that have been and are being applied in clinical practice. Among such targets is the enzyme dihydrofolate reductase (DHFR), a crucial enzyme in the folate metabolic pathway. This enzyme is responsible for reducing dihydrofolate (DHF) to its active form, tetrahydrofolate (THF). THF is a crucial cofactor required for the biosynthesis of purines, thymidylate, and several amino acids. THF is also required for the synthesis of DNA, RNA, and proteins. In microbial cells, disruption of THF regeneration rapidly inhibits cell growth and ultimately leads to cell death [10,11].

The structural features of mycobacterial dihydrofolate reductase (*Mtb*-DHFR) include an adenosine-binding subdomain and a flexible loop subdomain comprising the L1, L4, and L5 loops. The conformational changes in the loops play a crucial role in regulating the enzyme during the reduction of DHF to THF. Crystallographic studies have also revealed a small hydrophilic pocket adjacent to the folate-binding site in *Mtb*-DHFR, but not in human DHFR. This structural feature of the enzyme provides a promising avenue for the design of selective inhibitors for the treatment of tuberculosis.

Recent studies have also shown that alternative reductases such as RV2671 can partially compensate for DHFR activity, highlighting the central importance of THF regeneration for mycobacterial survival. Taken together, the essential role of DHFR in folate metabolism, its structural divergence from the human counterpart, and the availability of high-resolution crystal structures strongly support *Mtb*-DHFR as a rational and promising target for antitubercular drug discovery [12]. On this basis, the present study was undertaken to design and synthesize novel 5,6-diphenyl-1,2,4-triazine-piperazine derivatives and to evaluate their antitubercular potential as DHFR-targeted inhibitors.

The development of novel compounds based on medicinally relevant heterocycles is one of the most commonly employed methods in contemporary drug discovery. Among these is the class of nitrogen heterocycles, which have attracted researchers' interest due to their biological activities and potential.

In particular, heterocycles with bridgehead nitrogen atoms, such as (1,2,4,5)-tetrazine, (1,2,4)-triazole, and (1,2,4)-triazine derivatives, have attracted interest from drug researchers due to their potential for various biological and pharmaceutical applications. Among these is the 1,2,4-triazine nucleus, which is of interest for its potential in various biological and pharmaceutical applications, owing to its structural properties and stability in pharmacological interactions [13-15].

The derivatives of the 1,2,4-triazine ring system have been found to possess considerable biological activity. For example, lamotrigine, an anticonvulsant drug, and tirapazamine, an antitumor drug, are all derived from the triazine structure. In addition, fused 1,2,4-triazine derivatives have been found to possess potent antimicrobial, antiviral, and antimycobacterial activity. This unique structure of the 1,2,4-triazine ring system and its ability to interact with various biological pathways have led to further research into the development of drug molecules [16,17].

Piperazine, another nitrogen-heterocyclic compound, has also emerged as an important compound in medicinal chemistry due to its wide variety of pharmacological activities, such as antimycobacterial [18], antibacterial [19,20], antiviral [21], antifungal [22], antitumor [23], analgesic [24], anti-Alzheimer [25] and anticonvulsant activities [26]. The piperazine ring is recognized as a potential pharmacophore for the treatment of mycobacterial infections, offering enhanced hydrogen-bonding potential, dipole moment, stability, and rigidity in vivo [27]. These properties have been correlated to the enhanced pharmacological activities of the piperazine derivatives. This has also been attributed to the enhanced pharmacological activity of piperazine derivatives, which have been proposed as promising candidates for developing new anti-TB agents [28]. In addition, the presence of piperazine in the specific DprE1 inhibitor benzothiazinone (PBTZ169) has established piperazine as a promising compound [29,30].

**Figure 1.**
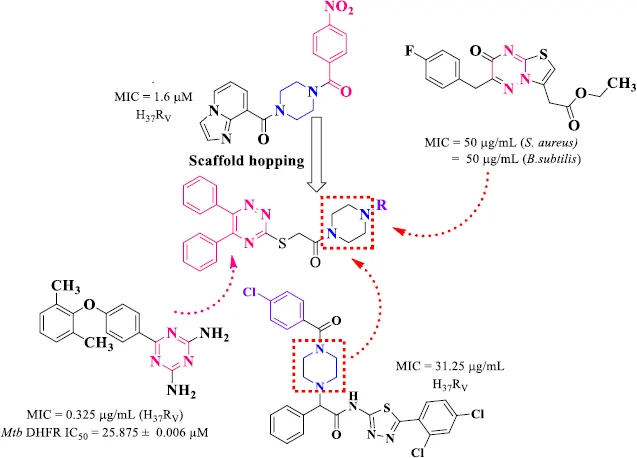
Designing strategy for 5,6-diphenyl-1,2,4-triazine-piperazine derivatives as potential anti-TB leads

In the last few years, the term ‘molecular hybridization’ (MH) has been recognized as a potent tool in medicinal chemistry. This method of drug design involves combining different pharmacophore groups of compounds to generate a single hybrid with the desired pharmacological activity [31,32]. The hybrids have been found to possess superior therapeutic activity and physicochemical characteristics, making them of considerable interest as potential drug candidates against DHFR inhibitors Figure 1 [33-35].

In view of the urgent need to develop novel and potent anti-tuberculosis agents, the search for novel molecular hybrids of 1,2,4-triazine and piperazine moieties presents an exciting research opportunity. Through the systematic synthesis of these compounds, it is possible to fully exploit their unique characteristics to develop potent antimycobacterial agents. This approach not only helps to resolve the challenges associated with the treatment of tuberculosis but also offers a pathway to the development of novel second-generation chemotherapeutic agents with a wide range of activity and reduced toxicity. As a part of our ongoing research towards the synthesis of novel biologically active 1,2,4-triazine derivatives, here we report the synthesis and pharmacological activity of novel 1-(4-benzoyl-piperazin-1-yl)-2-((5,6-diphenyl-1,2,4-triazin-3-yl)thio)ethan-1-one.

## Experimental

### In silico studies

#### Molecular docking studies

Molecular docking studies were conducted using ADT software to assess the interactions between the designed compounds and the key enzymes targeted in mycobacteria (DprE1 and *Mtb*-DHFR) The crystal structures of these enzymes were obtained from the RCSB Protein Data Bank (http://www.rcsb.org) with the following protein data bank (PDB) codes: 4NCR for DprE1 and 1DF7 for *Mtb*-DHFR, [36,37]. The ADT program prepared the enzyme and ligand structures in protein data bank, partial charge *Q* and atom type *T* (PDBQT) format by adding polar hydrogens, removing water molecules and applying Kollman charges. Grid boxes (60×60×60 Å) were created to define the active sites, with specific coordinates for each enzyme: DprE1 (center_x = 17.29, center_y = -20.91, center_z = -1.71) and *Mtb*-DHFR (center_x = 3.32, center_y = 27.33, center_z = 11.77). To validate the docking process, the co-crystallized ligands were removed and the enzymes were re-docked. The docking results and receptor-ligand interactions were analysed and visualized using Discovery Studio 2021 Client [38].

#### Molecular dynamics simulations

The highest-ranked docking conformations of the three top-performing compounds, together with a reference ligand, were further evaluated through molecular dynamics (MD) simulations to gain deeper insight into ligand-target interactions. All simulations were carried out using the Desmond v7.6 package developed by D. E. Shaw Research [39]. For each ligand-protein complex, a 200 ns MD simulation was executed following a standardized workflow comprising system preparation, energy minimization, and production dynamics. Each system was embedded in an orthorhombic simulation box and solvated with explicit TIP3P water molecules, maintaining a minimum distance of 0.10 nm between the solute and the box edges. Appropriate amounts of Na⁺ and Cl⁻ ions were introduced to neutralize the system and achieve a physiological ionic strength of 0.15 M. Prior to the production run, energy minimization was performed under isothermal-isobaric ensemble (NTP) ensemble conditions at 300 K and 100 kPa to eliminate unfavourable contacts. The MD simulations were conducted for 200 ns, with trajectory snapshots saved at 10 ps intervals, yielding a total of 20,000 frames *per* system. Following completion of the simulations, trajectory analyses were performed using the Simulation Interaction Diagram module of Desmond. Stability and dynamic behaviour of the ligand-protein complexes were assessed by calculating key parameters such as root mean square deviation (RMSD), root mean square fluctuation (RMSF), radius of gyration (*R*_g_) and protein-ligand interaction profiles throughout the simulation period [40].

#### Density function theory calculations

Density functional theory (DFT) calculations were carried out employing the hybrid B3LYP-D3 functional in conjunction with the 6-31G** basis set using the Jaguar v12.6 module of the Schrödinger Materials Suite [41]. The B3LYP functional is widely used owing to its reliable, balanced performance across a wide range of chemical systems. Inclusion of Grimme’s D3 dispersion correction [42] enhances the accuracy of modelling noncovalent interactions, conformational energetics, and reaction barriers. The polarized 6-31G** basis set provides a computationally efficient framework which, when combined with D3 (and optionally gCP), delivers qualitatively robust results suitable for preliminary geometry optimization and screening studies. Following geometry optimization, key electronic properties, including HOMO and LUMO energies, electrostatic potential, and related molecular parameters, were evaluated [43].

### Chemistry

All chemicals used for the synthesis were procured from Spectrochem Private Limited, Sigma-Aldrich and Avra Synthesis Private Limited. Prior to use, reagents and solvents were purified according to standard laboratory procedures. The progress of reactions was monitored by thin-layer chromatography (TLC) on pre-coated silica gel GF254 plates, and the spots were visualized under UV light at 254 or 365 nm. Elution was carried out using different solvent systems, including hexane-ethyl acetate (7:3 and 6:4) and dichloromethane-methanol (9:1 volume ratio). Solvent removal during work-up was performed using a BUCHI R-300 rotary evaporator. Purification of the crude products was achieved by column chromatography using silica gel (100to 200 mesh). Melting points of the synthesized compounds were determined using a Veego VMP-D digital melting point apparatus and are reported without correction. Infrared spectra were recorded on a Bruker ALPHA-FT-IR spectrophotometer equipped with an ATR accessory, and absorption bands are expressed in cm^-1^. Molecular mass determination was carried out using a Waters Acquity QDA mass spectrometer. ^1^H and ^13^C NMR spectra were acquired on a Bruker 400 MHz NMR spectrometer using CDCl₃ or DMSO-d₆ as solvents, with tetramethylsilane (TMS) as the internal reference. Elemental composition and purity of the synthesized compounds were confirmed by elemental analysis using a Thermo Fisher FLASH 2000 organic elemental analyser, with the obtained values lying within ±0.4 % of the calculated carbon, hydrogen, and nitrogen contents.

#### General method for acid-amine coupling (11 to 19)

To the solution of substituted benzoic acids (**2-10**) (1.0 g) in DMF (10 mL), EDC.HCl (1.1 equiv) and HOBt (1.1 equiv) were added, and the reaction mixture was stirred at a temperature between 5 and 10 °C for a time of 20 min. The 1-Boc-piperazine (1.0 equiv) was added to the above solution, followed by triethylamine (3 equiv). Stirring was continued at RT for 8-10 h and the progress of the reaction was monitored by TLC using (50 % ethyl acetate in hexane). After consumption of the starting materials, the reaction mixture was poured into ice-cold water to obtain solid products (**11**-**19**).

*Tert-butyl 4-benzoylpiperazine-1-carboxylate* (**11**): Unsing benzoic acid (**2**) (1.0 g, 8.1 mM) to obtain the desired product (**12**) as a white solid (1.65 g, 70 %), m.p. 187-189 °C, TLC (*R*_f_): 0.7 (50 % ethyl acetate in hexane), IR: 3004, 2977, 2928 2867, 1686, 1620, 1425, 1241, 1005 cm^-1^.

*Tert-butyl 4-(4-chlorobenzoyl)piperazine-1-carboxylate* (**12**): Unsing 4-chlorobenzoic acid (**3**) (1.0 g, 6.38 mM) to obtain the desired product (**13**) as a white solid (1.86 g, 90 %), melting point (m.p.) 183-186 °C, TLC (*R*_f_): 0.7 (50 % ethyl acetate in hexane), IR: 2967, 2930, 2846, 1681, 1621, 1439, 1242, 1158, 1116, 1077 cm^-1^.

*Tert-butyl 4-(4-fluorobenzoyl)piperazine-1-carboxylate* (**13**): Unsing 4-fluorobenzoic acid (**4**) (1.0 g, 7.13 mM) to obtain the desired product as a white solid (2.02 g, 92 %), m.p. 185-187 °C, TLC (*R*_f_): 0.67 (50 % ethyl acetate in hexane), IR: 3003, 2978, 2927, 1688, 1621, 1427, 1286, 1243, 1068 cm^-1^.

*Tert-butyl 4-(4-bromobenzoyl)piperazine-1-carboxylate* (**14**): Unsing and 4-bromobenzoic acid (**5**) (1.0 g, 4.97 mM) to obtain the desired product as a white solid (1.62 g, 89 %), m.p. 180-183 °C, TLC (*R*_f_): 0.77 (50 % ethyl acetate in hexane), IR: 3002, 2977, 2928, 1683, 1620, 1426, 1285, 1243, 1069, 1005 cm^-1^.

*Tert-butyl 4-(4-nitrobenzoyl)piperazine-1-carboxylate* (**15**): Unsing 4-nitrobenzoic acid (**6**) (1.0 g, 5.98 mM) to obtain the desired product as a yellow solid (1.84 g, 92 %), m.p. 176-179 °C, TLC (*R*_f_): 0.82 (50 % ethyl acetate in hexane), IR: 3002, 2979, 2927, 1688, 1624, 1427, 1285, 1244, 1064, 1006 cm^-1^.

*Tert-butyl 4-(4-methoxybenzoyl)piperazine-1-carboxylate* (**16**): Unsing 4-methoxybenzoic acid (**7**) (1.0 g, 6.57 mM) to obtain the desired product as a white solid (1.93 g, 92 %), m.p. 189-192 °C, TLC (*R*_f_): 0.75 (50 % ethyl acetate in hexane), IR: 3002, 2979, 2927, 1685, 1624, 1422, 1285, 1246, 1067, 1004 cm^-1^.

*Tert-butyl 4-(2-chlorobenzoyl)piperazine-1-carboxylate* (**17**): Unsing 2-chlorobenzoic acid (**8**) (1.0 g, 6.38 mM) water to obtain the desired product as a white solid (1.84 g, 89 %), m.p. 179-182 °C, TLC (*R*_f_): 0.69 (50 % ethyl acetate in hexane), IR: 2967, 2930, 1681, 1621, 1483, 1287, 1242, 1053, 1007 cm^-1^.

*Tert-butyl 4-(4-methylbenzoyl)piperazine-1-carboxylate* (**18**): Unsing 4-methylbenzoic acid (**9**) (1.0 g, 7.34 mM) to obtain the desired product as a white solid (1.98 g, 89 %), m.p. 185-188 °C, TLC (*R*_f_): 0.83 (50 % ethyl acetate in hexane), IR: 3002, 2979, 2929, 1687, 1625, 1456, 1423, 1285, 1245, 1120, 1006 cm^-1^.

*Tert-butyl 4-(2,4-dichlorobenzoyl)piperazine-1-carboxylate* **(19):** Unsing 2,4-dicholobenzoic acid (**10**) (1.0 g, 5.20 mM) to obtain the desired product as a white solid (1.81 g, 87 %), m.p. 185-187 °C, TLC (R_f_): 0.82 (50 % ethyl acetate in hexane), IR: 2967, 2930, 1681, 1621, 1439, 1408, 1287, 1242, 1116, 1007 cm^-1^.

#### General method for Boc-deprotection to obtain 20 to 28 (Method B)

To the solution of corresponding products (**11** to **19**) in DCM (7.5 mL), dioxane HCl (7.5 mL) was added and stirred at 25 °C for 3 h. The reaction was monitored by TLC after completion; the reaction mixture was then removed under reduced pressure to obtain the solid product (**20** to **28**).

*Phenyl(piperazin-1-yl)methanone* (**20**): *tert-butyl 4-benzoylpiperazine-1-carboxylate* (**10**) (1.5 g, 5.16 mM) offered the compound **(20)** (0.93 g, 95 %), which was further processed for the next step of the reaction. TLC (*R*_f_): 0.30 (10 % methanol in dichloromethane).

*(4-chlorophenyl)(piperazin-1-yl)methanone* (**21**): *tert-butyl 4-(4-chlorobenzoyl)piperazine-1-carboxylate* (**11**) (1.5 g, 4.61 mM) offered the compound (**21**) (1.03 g, 98 %), which was further processed for next step of the reaction. TLC (*R*_f_): 0.28 (10 % methanol in dichloromethane).

*(4-fluorophenyl)(piperazin-1-yl)methanone* (**22**): *tert-butyl 4-(4-fluorobenzoyl)piperazine-1-carboxylate* (**12**) (1.5 g, 4.86 mM) offered the compound **(22)** (0.98 g, 97 %), which was further processed for next step of the reaction. TLC (*R*_f_): 0.26 (10 % methanol in dichloromethane).

*4-bromophenyl)(piperazin-1-yl)methanone* (**23**): *tert-butyl 4-(4-bromobenzoyl)piperazine-1-carboxylate* (**13**) (1.5 g, 4.06 mM) offered the compound **(23)** (1.03 g, 95 %) was further processed for next step of the reaction. TLC (*R*_f_): 0.29 (10 % methanol in dichloromethane).

*(4-nitrophenyl)(piperazin-1-yl)methanone* (**24**): *tert-butyl 4-(4-nitrobenzoyl)piperazine-1-carboxylate* (**14**) offered the compound **(24)** (0.99 g, 95 %) was further processed for the next step of the reaction. TLC (*R*_f_): 0.31 (10 % methanol in dichloromethane).

*(4-methoxyphenyl)(piperazin-1-yl)methanone* (**25**): *tert-butyl 4-(4-methoxybenzoyl)piperazine-1-carboxylate* (**15**) (1.5 g, 4.68 mM) offered the compound **(25)** (1.0 g, 97 %) was further processed for next step of the reaction. TLC (*R*_f_): 0.32 (10 % Methanol in dichloromethane).

(*2-chlorophenyl)(piperazin-1-yl)methanone* (**26**): *tert-butyl 4-(2-chlorobenzoyl)piperazine-1-carboxylate* (**16**) (1.5 g, 4.61 mM) offered the compound **(26)** (0.97 g, 94 %), which was further processed for next step of the reaction. TLC (*R*_f_): 0.31 (10 % methanol in dichloromethane).

*Piperazin-1-yl(p-tolyl)methanone* (**27**): *tert-butyl 4-(4-methylbenzoyl)piperazine-1-carboxylate* (**17**) (1.5 g, 4.92 mM) offered the compound (**27**) (0.96 g, 96 %) was further processed for the next step of the reaction. TLC (*R*_f_): 0.27 (10 % methanol in dichloromethane).

(2,4-dichlorophenyl)(piperazin-1-yl)methanone (**28**): *tert-butyl 4-(2,4-dichlorobenzoyl)piperazine-1-carboxylate* **(19)** (1.5 g, 4.82 mM) offered the compound (**28**) (0.97 g, 95 %) was further processed for the next step of the reaction. TLC (*R*_f_): 0.23 (10 % methanol in dichloromethane).

#### General method for chloro-amine coupling (29 to 37) (Method C)

To the solution of corresponding products (**20** to **28**) (1.0 g) in DCM (10 mL), triethylamine (1.5 equiv) was added. and the reaction mixture was stirred at ice-cold conditions at 0 °C. Chloroacetyl chloride (1 equiv) was added dropwise, and the reaction was allowed to proceed at room temperature for 4 to 5 h. The progress of the reaction was monitored by TLC using (60 % ethyl acetate in hexane). After the consumption of starting materials, the resulting residue was washed with water and extracted in DCM. The organic layer was removed under reduced pressure to obtain the desired product (**29** to **37)**.

*1-(4-benzoylpiperazin-1-yl)-2-chloroethan-1-one* (**29**): Using *phenyl(piperazin-1-yl)methanone* (**20**) (0.85 g, 4.46 mM) to obtained **(29)** as - solid (0.95 g, 80 %), m.p. 149-151 °C, TLC (*R*_f_): 0.37 (70 % ethyl acetate in hexane), IR: 2924, 2863, 1629, 1509, 1426, 1223, 1005, 847 cm^-1^.

*2-chloro-1-(4-(4-chlorobenzoyl)piperazin-1-yl)ethan-1-one* (**30**): Using *(4-chlorophenyl)(piperazin-1-yl)methanone* (**21**) (0.85 g, 3.78 mM) to obtained **(30)** as - solid (0.96 g, 85 %), m.p. 165-168 °C, TLC (*R*_f_): 0.37 (70 % ethyl acetate in hexane), IR: 2924, 2866, 1743, 1632, 1605, 1430, 1246, 1194, 1004 744 cm^-1^.

*2-chloro-1-(4-(4-fluorobenzoyl)piperazin-1-yl)ethan-1-*one (**31**): Using *(4-fluorophenyl)(piperazin-1-yl)methanone* (**22**) (0.85 g, 3.78 mM) to obtained **(31)** (0.99 g, 86 %), m.p. 149-151 °C, TLC (*R*_f_): 0.49 (70 % ethyl acetate in hexane), IR: 2924, 2865, 1740, 1626, 1427, 1268, 1006, 788 cm^-1^.

*1-(4-(4-bromobenzoyl)piperazin-1-yl)-2-chloroethan-1-one* (**32**): Using *(4-bromophenyl)(piperazin-1-yl)methanone* (**23**) (0.85 g, 3.15 mM) to obtained **(32**) (0.96 g, 88 %), m.p. 149-151°C, TLC (*R*_f_): 0.39 (70 % ethyl acetate in hexane), IR: 2965, 2925, 1630, 1427, 1266, 1148, 1003, 834 cm^-1^.

*2-chloro-1-(4-(4-methoxybenzoyl)piperazin-1-yl)ethan-1-one* (**33**): Using *(4-methoxyphenyl)(piperazin-1-yl)methanone* (**24**) (0.85 g, 3.85 mM) to obtained **(33)** (0.98 g, 86 %), m.p. 153-157 °C, TLC (*R*_f_): 0.31 (70 % ethyl acetate in hexane), IR: 2928, 2859, 1626, 1512, 1425, 1248, 1173, 1001, 841 cm^-1^.

*2-chloro-1-(4-(4-nitrobenzoyl)piperazin-1-yl)ethan-1-one* (**34**): Using *(4-nitrophenyl)(piperazin-1-yl)methanone* (**25**) (0.85 g, 3.61 mM) to obtained **(34)** (0.97 g, 87 %), m.p. 144-147 °C, TLC (*R*_f_): 0.43 (70 % ethyl acetate in hexane), IR: 2996, 2922, 2361, 1634, 1521, 1433, 1349, 1152, 1004, 848 cm^-1^.

*2-chloro-1-(4-(2-chlorobenzoyl)piperazin-1-yl)ethan-1-one* (**35**): Using *(2-chlorophenyl)(piperazin-1-yl)methanone* (**26**) (0.85 g, 3.78 mM) to obtained **(35)** (0.94 g, 83 %), m.p. 150-153 °C, TLC (*R*_f_): 0.38 (70 % ethyl acetate in hexane), IR: 2924, 1742, 1633, 1481, 1431, 1284, 1194, 1051, 1004, 740 cm^-1^.

*2-chloro-1-(4-(4-methylbenzoyl)piperazin-1-yl)ethan-1-one* (**36**): Using *piperazin-1-yl(p-tolyl)methanone* (**27**) (0.85 g, 4.16 mM) to obtained (**36**) (1.0 g, 86 %), m.p. 175-178 °C, TLC (*R*_f_): 0.3 (70 % ethyl acetate in hexane), IR: 2921, 2864, 1632, 1430, 1274, 1147, 1005, 831 cm^-1^.

*3-chloro-1-(4-(2,4-dichlorobenzoyl)piperazin-1-yl)propan-1-one* (**37**): Using *(2,4-dichlorophenyl) (piperazin- -1-yl) methanone* (**28**) (0.85 g, 4.16 mM) to obtained (**37**) (0.93 g, 88 %), m.p. 185-187 °C, TLC (*R*_f_): 0.39 (70 % ethyl acetate in hexane), IR: 2924, 2865, 1742, 1632, 1430, 1284, 1030 cm^-1^.

#### General procedure for the synthesis of 5,6-diphenyl-1,2,4-triazine-3-thiol (38)

The compound (**38**) was prepared according to the reported procedure [44,45]. Briefly, a solution of benzil (1.0 g, 1 mM) in glacial acetic acid (10 mL) was stirred at 100 °C for 1 h. After that thiosemicarbazide (0.86 g, 9.6 mM) was added, the reaction was refluxed for 2-3 h, the progress of the reaction was monitored by TLC using (20 % ethyl acetate in hexane) After the consumption of starting materials, the reaction mixture cooled down and filter out to get orange precipitate and washed with cold acetic acid and water and recrystallized it form ethanol to get desired product (**34**). Yield: 85 %; m.p. 234 to 235 °C (m.p. 234 to 236 °C [44])

#### General procedure for the synthesis of the target compounds (FP1 to FP8) (Method D)

To the solution of compound (**38**) (1 equiv) in DMF (10 V), K_2_CO_3_ (1 equiv) was added. The reaction mixture was stirred at room temperature for 1 h. After that, the corresponding product (**29** to **37**) (1.2 equiv) and potassium iodide (0.6 equiv) were added to the above solution. The progress of the reaction was monitored by TLC using (60 % ethyl acetate in hexane). After consumption of the starting materials, the reaction mixture was poured into ice-cold water to obtain solid products (FP1-**FP9**), which were further purified by column chromatography using 100-200 mesh silica gel as the stationary phase and Ethyl acetate:hexane as the mobile phase to afford the desired pure products (**FP1** to **FP9**).

*1-(4-Benzoylpiperazin-1-yl)-2-((5,6-diphenyl-1,2,4-triazin-3-yl)thio)ethan-1-one* (**FP1**): Using *1-(4-benzoylpiperazin-1-yl)-2-chloroethan-1-one* (**26**) the desired compound (**FP1**) was obtained as a white solid (0.72 g, 60 %), which was further purified by column chromatography using 100-200 silica gel as stationary phase and ethyl acetate: hexane as mobile phase, m.p. 173-176 °C. TLC (*R*_f_): 0.43 (70 % ethyl acetate in hexane); IR: 2916, 1620, 1429, 1287,1258, 1184, 696 cm^-1^; 1H NMR: *δ* 7.49-7.45 (t, *J* = 9.3 Hz, 11H, ArH), 7.42 (s, 2H, ArH), 7.40-7.36 (d, *J* = 8.0 Hz, 2H, ArH), 4.45 (s, 2H, CH2), 3.64 (m, 8H, CH2); C_28_H_25_N_5_O_2_S requires: C, 67.86; H, 5.08; N, 14.13; found requires: C, 67.76; H, 5.03; N, 14.08; mass (*m*/*z*): 496.2 (M+H)^+^.

1-(4-(4-Chlorobenzoyl)piperazin-1-yl)-2-((5,6-diphenyl-1,2,4-triazin-3-yl)thio)ethan-1-one (**FP2**): Using *2-chloro-1-(4-(4-chlorobenzoyl)piperazin-1-yl)ethan-1-one* (**27**) the desired compound (**FP2**) was obtained as a light orange solid (0.81 g, 63 %), which was further purified by column chromatography using 100-200 silica gel as stationary phase and ethyl acetate: Hexane as mobile phase, m.p. 177-79 °C; TLC (*R*_f_): 0.47 (70 % ethyl acetate in hexane); IR: 2918, 2859, 1629, 1426, 1263, 1180, 1088, 754 cm^-1^; ^1^H NMR: *δ* 7.55-7.52 (d, *J* = 8.5 Hz, 2H, Ar*H*), 7.46-7.36 (m, 12H, ArH), 4.45 (s, 2H, CH_2_), 3.71-3.57 (m, 8H, CH_2_); C_28_H_24_ClN_5_O_2_S Rrequires: C, 63.45; H, 4.56; N, 13.21; found requires: C, 63.35; H, 4.47; N, 13.18; mass (*m*/*z*): 531.2 (M+H)^+^, 532.4 (M+2); HR-MS (*m*/*z*): [M+H]^+^ calculated 530.1373; found 530.1389.

*2-((5,6-Diphenyl-1,2,4-triazin-3-yl)thio)-1-(4-(4-fluorobenzoyl)piperazin-1-yl)ethan-1-one* (**FP3**): Using *2-chloro-1-(4-(4-fluorobenzoyl)piperazin-1-yl)ethan-1-one* (**28**) the desired compound (**FP 3**) was obtained as a light orange solid (0.80 g, 64 %), which was further purified by column chromatography using 100-200 silica gel as stationary phase and ethyl acetate: Hexane as mobile phase, m.p. 172-75 °C; TLC (R_f_): 0.52 (70 % ethyl acetate in hexane); IR: 2916, 2858, 1629, 1424, 1333, 1180, 1000, 755, 695 cm^-1^; ^1^H NMR: *δ* 7.70-7.67 (m, 2H, ArH), 7.49-7.36 (m, 12H, ArH), 4.45 (s, 2H, CH_2_), 3.70-3.57 (m, 8H, CH_2_); C_28_H_24_FN_5_O_2_S Requires: C, 65.48; H, 4.71; N, 13.64; found requires: C, 65.38; H, 4.68; N, 13.59; Mass (*m*/*z*): 514.3 (M+H)^+^; HR-MS (*m*/*z*): [M+H]^+^ calculated 514.1668; found 514.1684.

*1-(4-(4-Bromobenzoyl)piperazin-1-yl)-2-((5,6-diphenyl-1,2,4-triazin-3-yl)thio)ethan-1-one* (**FP4**): Using 1*-(4-(4-bromobenzoyl)piperazin-1-yl)-2-chloroethan-1-one* (**29**) the desired compound (**FP4**) was obtained as a light yellow solid (0.97 g, 69 %), which was further purified by column chromatography using 100-200 silica gel as stationary phase and ethyl acetate: Hexane as mobile phase, m.p. 175-78 °C; TLC (R_f_): 0.46 (70 % ethyl acetate in hexane); IR: 2920, 2859, 1633, 1427, 1334, 1182, 1000, 757, 696 cm*^-^*^1^; ^1^H NMR: *δ* 7.54-7.29 (m, 14H, ArH), 4.44 (s, 2H, CH_2_), 3.69-3.34 (m, 8H, CH_2_); C_28_H_24_BrN_5_O_2_S Requires: C, 58.54; H, 4.21; N, 12.19; found requires: C, 58.50; H, 4.18; N, 12.22; mass (*m*/*z*): 576.0 (M+2H)^+^.

*2-((5,6-diphenyl-1,2,4-triazin-3-yl)thio)-1-(4-(4-nitrobenzoyl)piperazin-1-yl)ethan-1-one* (**FP5**): Using that *2-chloro-1-(4-(4-nitrobenzoyl)piperazin-1-yl)ethan-1-one* (**30**) the desired compound (**FP5**) was obtained as a light yellow solid (0.86 g, 65 %), which was further purified by column chromatography using 100-200 silica gel as stationary phase and ethyl acetate: Hexane as mobile phase, m.p. 176-79 °C; TLC (*R*_f_): 0.46 (70 % ethyl acetate in hexane); IR: 2956, 2923, 1632, 1514, 1427, 1336, 1177, 1004, 696 cm^-1^; 1H NMR: *δ* 8.33-8.31 (d, *J* = 8.4 Hz, 2H, ArH), 7.74-.72 (d, *J* = 8.5 Hz, 2H, ArH), 7.49-7.39 (m, 10H, ArH), 4.49-4.41 (d, *J* = 30.4 Hz, 2H, CH_2_), 3.76-3.51 (t, *J* = 49.2 Hz, 6H, CH_2_), 3.35-3.25 (d, *J* = 42.1 Hz, 2H, CH_2_); C_28_H_24_N_6_O_4_S requires: C, 62.21; H, 4.48; N, 15.22; found requires: C, 62.18; H, 4.40; N, 15.18; mass (*m*/*z*): 541.4 (M+H)^+^.

*2-((5,6-diphenyl-1,2,4-triazin-3-yl)thio)-1-(4-(4-methoxybenzoyl)piperazin-1-yl)ethan-1-one* (**FP6**): Using *2-chloro-1-(4-(4-methoxybenzoyl)piperazin-1-yl)ethan-1-one* (**31**) the desired compound (**FP 6**) was obtained as a white solid (0.87 g, 68 %), which was further purified by column chromatography using 100-200 silica gel as stationary phase and ethyl acetate: Hexane as mobile phase, m.p. 178-81°C; TLC (*R*_f_): 0.43 (70 % ethyl acetate in hexane); IR: 2955, 2922, 1630, 1425, 1335, 1174, 1002, 758, 696 cm^-1^; ^1^H NMR: δ 7.50-7.36 (m, 12H, ArH), 7.03-7.00 (m, 2H, ArH), 4.44 (s, 2H, CH_2_), 3.81 (s, 3H, OCH_3_), 3.68-3.48 (m 8H, C*H*_2_); C_29_H_27_N_5_O_3_S requires: C, 66.27; H, 5.18; N, 13.32; found requires: C, 66.20; H, 5.16; N, 13.28; mass (*m*/*z*): 526.2(M+H)^+^.

*1-(4-(2-Chlorobenzoyl)piperazin-1-yl)-2-((5,6-diphenyl-1,2,4-triazin-3-yl)thio)ethan-1-one* (**FP7**): Using 2*-chloro-1-(4-(2-chlorobenzoyl)piperazin-1-yl)ethan-1-one* (**32**) the desired compound (**FP 7**) was obtained as a light orange solid (0.89 g, 69 %), which was further purified by column chromatography using 100-200 silica gel as stationary phase and ethyl acetate: Hexane as mobile phase, m.p. 174-77 °C; TLC (*R*_f_): 0.42 (70 % ethyl acetate in hexane); IR: 2922, 1631, 1482, 1336, 1177, 1002, 696 cm^-1^; ^1^H NMR: *δ* 8.34-8.27 (m, 2H, ArH), 7.96-7.90 (d, *J* = 16.9 Hz, 1H, ArH), 7.80-7.76 (t, *J* = 7.9 Hz, 1H, ArH), 7.55-7.39 (m, 11H, ArH), 4.49-4.40 (d, *J* = 25.8 Hz, 2H, CH_2_), 3.76-3.40 (m, 8H, CH_2_); C_28_H_24_ClN_5_O_2_S Requires: C, 63.45; H, 4.56; N, 13.21; Found Requires: C, 63.40; H, 4.50; N, 13.16; mass (*m*/*z*): 530.3 (M+).

*2-((5,6-diphenyl-1,2,4-triazin-3-yl)thio)-1-(4-(4-methylbenzoyl)piperazin-1-yl)ethan-1-one* (**FP8**): Using *2-chloro-1-(4-(4-methylbenzoyl)piperazin-1-yl)ethan-1-one* (**33**) the desired compound (**FP8**) was obtained as a white solid (0.87 g, 70 %), which was further purified by column chromatography using 100-200 silica gel as stationary phase and ethyl acetate: hexane as mobile phase, m.p.173-76 °C; TLC (*R*_f_): 0.43 (70 % ethyl acetate in hexane); IR: 2958, 2921, 1632, 1429, 1363, 1178, 1004, 766, 696 cm^-1^; ^1^H NMR: *δ* 7.49-7.26 (m, 14H, ArH), 4.44 (s, 2H, CH_2_), 3.68-3.36 (m, 8H, CH_2_), 2.35 (s, 3H, CH_3_); C_29_H_27_N_5_O_2_S requires: C, 68.35; H, 5.34; N, 13.74; found requires: C, 68.30; H, 5.30; N, 13.70; mass (*m*/*z*): 510.4 (M+H)^+^; HR-MS (*m*/*z*): [M+H]^+^ calculated 510.1919; found 510.1946.

*1-(4-(2,4-dichlorobenzoyl)piperazin-1-yl)-2-((5,6-diphenyl-1,2,4-triazin-3-yl)thio)ethan-1-one* **(FP9):** Using 2-chloro-1-(4-(2,4-dichlorobenzoyl)piperazin-1-yl)ethan-1-one (34) the desired compound (**FP9**) was obtained as a white solid (0.82 g, 69 %), which was further purified by column chromatography using 100-200 silica gel as stationary phase and ethyl acetate: Hexane as mobile phase, m.p.171-73 °C; TLC (*R*_f_): 0.41 (70 % ethyl acetate in hexane); IR: 2958, 2921, 1632, 1429, 1363, 1178, 1004, 766, 696 cm^-1^; ^1^H NMR: *δ* 7.78-7.75 (dd, *J* = 6.5, 2.0 Hz, 1H, ArH), 7.57-7.33 (m, 13H, ArH), 4.50-4.37 (m, 2H, CH_2_), 3.74 (s, 2H, CH_2_), 3.60 (m, 2H, CH_2_), 3.48-3.41 (m, 2H, CH_2_), 3.19-3.10 (m, 2H, CH_2_); C_28_H_23_N_5_O_2_S requires: C, 59.58; H, 4.11; N, 12.14; found requires: C, 59.48; H, 4.04; N, 12.11; mass (*m*/*z*): 565.3 (M+H)^+^

### Biological Activity

#### Anti-tubercular activity

The synthesized analogues (**FP1** to **FP9**) were screened in vitro for their anti-mycobacterial activity against the H37Rv strain using the Microplate Alamar Blue Assay method [46]. The reference standard drugs, such as isoniazid, ethambutol, and pyrazinamide, were also used for comparison. The steps involved in the Microplate Alamar Blue Assay method for screening the anti-mycobacterial activity of the synthesized analogues (**FP1** to **FP9**) are as follows: 200 μL of sterile deionized water was added to all the outer perimeter wells of the sterile 96-well plate to prevent the evaporation of the medium in the wells during the incubation period. The 96-well plate was then supplemented with 100 μL of Middlebrook 7H9 broth, and the serial dilutions of the test drugs were directly prepared on the plate. The concentration range of the test drugs was maintained between 100 and 0.2 μg mL^-1^. The plates were covered, sealed with parafilm, and incubated for five days at 37 °C. After the incubation period, 25 μL of the freshly prepared mixture of Alamar Blue reagent and Tween 80 (10 wt.%) was added to the plate, followed by further incubation for 24 hours. The colour change in the wells, *i.e.* blue colour indicating the absence of growth, and pink colour indicating the presence of growth, was recorded, and the minimum inhibitory concentration was determined.

#### *In vitro* cytotoxicity assay

The cytotoxic potential of the test samples was studied on the RAW 264.7 cell line obtained from the National Centre for Cell Science (Pune). The MTT assay was used for the purpose of the experiment. Cells were seeded at a density of 10^4^ cells *per* well in a 96-well plate and allowed to adhere for 24 h in DMEM medium supplemented with 10 % foetal bovine serum and 1 % penicillin-streptomycin solution. The cell cultures were maintained at 37° C in a humidified incubator with 5 % CO_2_ supplementation. After 24 h of cell seeding, the cells were treated with different concentrations of the test compounds. The stock solution of the test compounds was prepared in dimethyl sulfoxide and then diluted with incomplete medium to obtain the required concentrations. After 24 h of treatment of the cells with the test compounds, MTT solution (5 μg mL^-1^) was added to each well of the plate and incubated for a period of 2 h in a CO_2_ incubator. The wells containing untreated cells were considered the control, while the wells devoid of cells were considered the blank. After the experiment was completed, the medium was carefully discarded, and the resulting crystals were dissolved in 100 μL of DMSO. The absorbance was measured at 540 nm using an ELISA microplate reader (iMark Bio-Rad USA). The IC_50_ values for the compounds were obtained using GraphPad Prism software version 6 [47-48].

#### *In silico* drug-likeness and ADMET prediction studies

The pharmacokinetic profiles and drug-likeness of the studied compounds were also predicted using the SWISSADME tool. This tool was used to investigate key physicochemical parameters of the studied compounds, including molecular weight, the number of hydrogen-bond donors and acceptors, the n-octanol/water partition coefficient (log *P*), and the number of rotatable bonds. Other important parameters related to absorption, distribution, metabolism, and excretion were also studied using the same tool [49].

## Result and discussion

### Chemistry

**Scheme 1.**
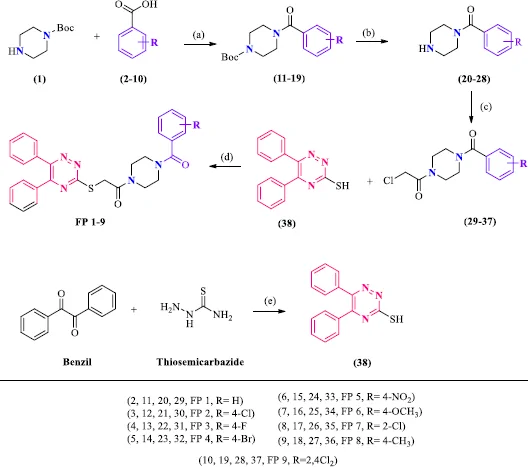
Synthesis of 5,6-diphenyl-1,2,4-triazine-piperazine derivatives (**FP1** to **FP9**). Reagents and conditions: (a) EDC.HCl, HOBt, TEA, DMF, r.t. 8-10 h; (b) Dioxane. HCl, DCM, r.t., 5-6 h (c) chloroacetyl chloride, DIPEA, DCM, r.t., 4-5 h; (d) K_2_CO_3_, KI, DMF, 80 °C, 8 h (e) Glacial acetic acid, reflux, 2-3 h

To synthesize 5,6-diphenyl-1,2,4-triazine-piperazine derivatives, a synthetic route has been optimized, as depicted in Scheme 1.

In the first step, *N*-Boc-piperazine (**1**) was reacted with various substituted benzoic acids (**2** to **10**) in the presence of coupling agents EDC·HCl and HOBt, using triethylamine as a base in DMF, to afford the corresponding amides (**11** to **19**). Subsequent deprotection of the Boc group using HCl in dioxane yielded the acetamides (**20** to **28**). In the next step, these acetamides (**20** to **28**) were coupled with chloroacetyl chloride in the presence of *N,N*-diisopropylethylamine to give the acetylated intermediates (**29** to **37**). In parallel, thiosemicarbazide reacted with benzil in glacial acetic acid to form intermediate (**38**), which was subsequently reacted in the final step with the acetylated intermediates (**29** to **37**) in the presence of potassium iodide and potassium carbonate in DMF to afford the final products (**FP1** to **FP9**). The synthesized target compounds (**FP1** to **FP9**) were characterized using spectral and elemental analyses.

### Molecular docking studies

Molecular modelling studies were carried out for the designed compounds, as well as the standard compounds, to justify the rationale behind the design of the hybrid compounds. As the exact site of action of the synthesized compounds was not identified, molecular docking studies were carried out for the synthesized compounds targeting two major enzymes of *Mycobacterium tuberculosis*, *i.e.* DprE1 and *Mtb*-DHFR. The docking analysis was performed to predict potential modes of action for the designed compounds. The docking analysis was performed to elucidate the interactions between the designed compounds and the active sites of the targeted enzymes.

**Figure 2.**
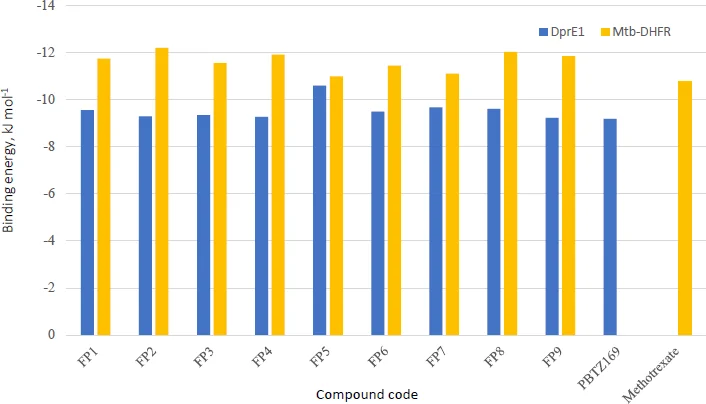
Molecular docking scores of the designed compounds (**FP1** to **FP9**)

Figure 2 presents the docking results for the synthesized compounds (**8** to **14**), as well as the standard inhibitors, *i.e.* PBTZ169 and methotrexate, to predict the binding affinities of the compounds with the targeted enzymes, *i.e.* DprE1 and *Mtb*-DHFR. The docking analysis was carried out using AutoDock Tools 1.5.7 [50], which estimated the binding energy between the ligand and the target proteins.

**Figure 3.**
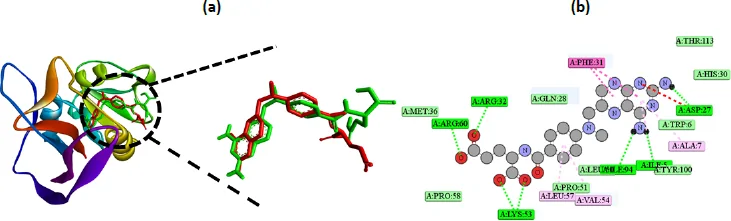
(a) validation results of the binding modes of methotrexate obtained using the AutoDock software, (b) docking conformation and *Mtb*-DHFR protein-ligand interactions of reference drug methotrexate

Figure 3 presents the results obtained for the docking analysis, which indicate the alignment of the proposed binding mode of the inhibitor with the active site of the targeted enzyme, *i.e. Mtb*-DHFR. The docking results were compared with the docking results of the standard inhibitor, *i.e.* methotrexate, which produced a root mean square deviation (RMSD) of 0.107 nm. The results were well within the acceptable range of 0.2 nm, which indicated the accurate docking results.

In the present study, the synthesized compounds were docked into the DprE1 (PDB ID: 4NCR) and *Mtb*-DHFR (PDB ID: 1DF7) proteins to assess their binding affinity for the receptors. All the designed molecules were docked into the same active site, wherein the co-crystallized ligand was lodged. It was observed that the inbuilt ligands PBTZ169 and Methotrexate showed binding affinity -38.45 and -45.19 kJ mol^-1^ (1 kJ = 0.239 kcal) towards the receptor, respectively. From that PBTZ169 that formed H, binding with amino acids like Lys134, Gly177, Gln336, CYS387, which are important amino acids. It was observed that CYS387, which is also involved in π-sulphur type of interaction and other amino acids, His132 with π-Cation, and Arg58, Trp16, Tyr60, Val365, Lys367 with Pi-Alkyl type of interaction was observed [51]. Designed compounds that showed binding affinity towards DprE1 ranged from -38.83 to -44.18 kJ mol^-1^ and -46.02 to -50.38 kJ mol^-1^ towards *Mtb*-DHFR. Based on the results, all compounds showed higher binding affinity for *Mtb*-DHFR than for DprE1, so a detailed molecular docking study is discussed here.

**Figure 4.**
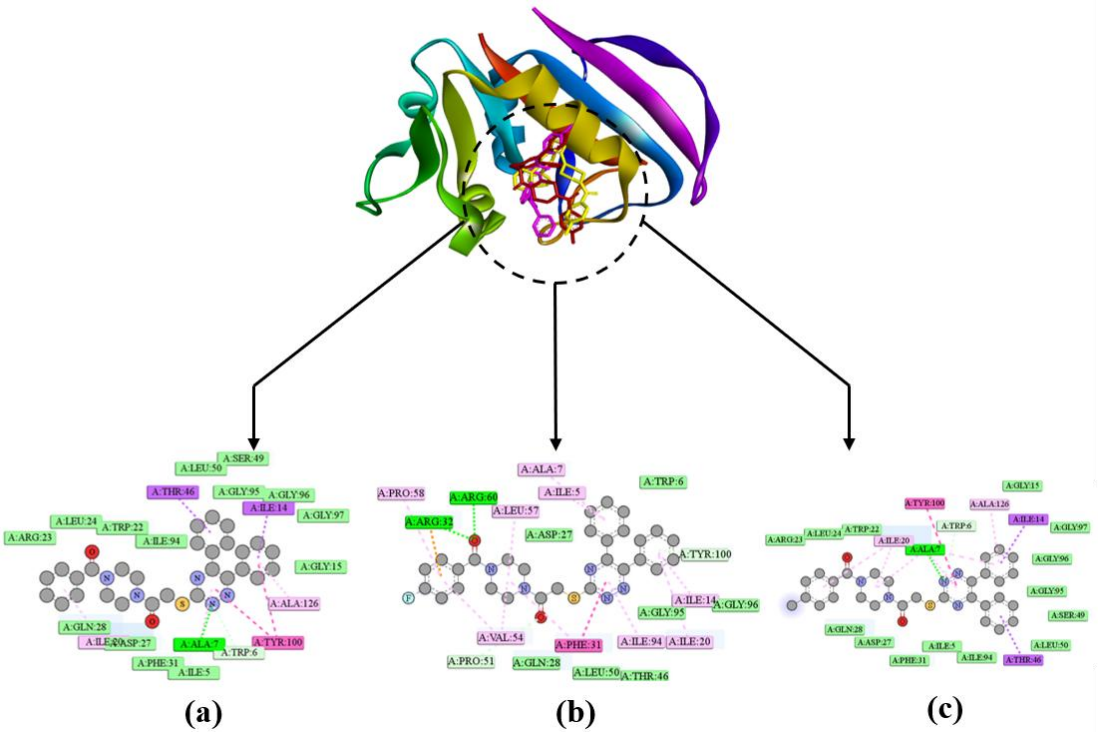
Docking conformations and *Mtb*-DHFR protein-ligand interactions of designed molecules. (a) **FP1**, (b) **FP3**, (c) **FP8**. Dark green colour indicates conventional hydrogen bonding, light green colour indicates Van der Waals interactions, purple colour indicates π-sigma interactions, pink colour indicates hydrophobic interactions (π-π stacking, π-π T-shaped)

Compound **FP1**, which had a binding affinity of -49.16 kJ mol^-1^ towards *Mtb*-DHFR, formed hydrogen bonding with the Ala7 amino acid and π-sigma type of interaction with Ile14 and Thr46 amino acids. The phenyl ring of the structure also formed a π-alkyl interaction with Ile20. The docked conformation **FP3** showed that the ligand formed key stabilizing interactions within the active site (Figure 4).

The triazine ring engaged in π-π stacking with Phe31 and π-alkyl interactions with Ile14, whereas the aromatic ring formed interactions with Ile20 and Ile94 residues. The carbonyl oxygen atom of the compound is engaged in hydrogen bonding with Arg32 and Arg60. The piperazine ring formed an alkyl interaction with Leu57 and Val54, with a binding affinity of -48.37 kJ mol^-1^ towards *Mtb*-DHFR. Compound **FP3** also has a binding affinity of -39.12 kJ mol^-1^ towards DprE1 and forms hydrogen bonding with Tyr60 and Lys134 amino acids. Compound **FP4**, **FP5**, **FP6**, **FP7** and **FP9** that formed a common type of interaction with Ala7, Ile20, Arg32, and Thr46 amino acid, and having binding affinity -49.87, -46.02, -47.91, -46.48, -49.62 and -46.65 kJ mol^-1^, respectively. Meanwhile, compound **FP8**, which has a binding affinity of -50.38 kJ mol^-1^ and a triazine ring that forms hydrogen bonding with Ala7 and pi-pi T-shaped interaction with Tyr100, a further biphenyl ring that forms π-sigma type interaction with Ile14, and Thr46 amino acid. This compound **FP8** also exhibited a binding affinity of -9.62 towards DprE1 and interacted with key amino acids such as Pro116 (π-π), Lys134 (π-Alkyl), Leu363 (π-Alkyl), Val365 (π-Alkyl), Lys367 (π-Alkyl), Cys387 (π-s), and Tyr415 (π-Cation).

### Molecular dynamics simulation

**Figure 5.**
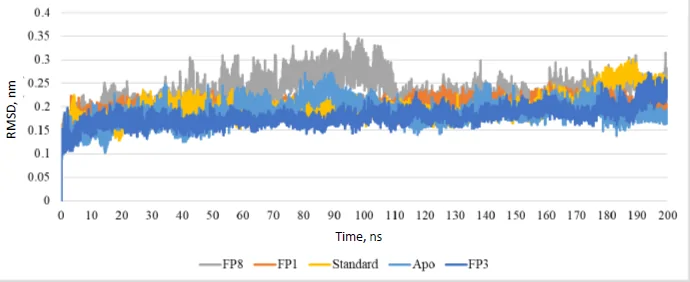
RMSD of protein backbone on binding of the ligands (reference, **FP1**, **FP3**, **FP8** and apo) to the target enzyme *Mtb*-DHFR

Following molecular docking, the stability and dynamic behaviour of the protein-ligand complexes were examined through molecular dynamics simulations. The backbone RMSD and RMSF parameters were used to assess structural deviations and flexibility relative to the unbound protein. The RMSD profiles of the protein in its apo form and in complex with the reference molecule, as well as **FP1**, **FP3** and **FP8** ligands, are shown in Figure 5. The apo structure exhibited greater fluctuations throughout the simulation, indicating a more flexible conformation in the absence of a bound ligand. In contrast, the ligand-bound systems exhibited quicker stabilization during the initial equilibration phase, followed by relatively stable RMSD trajectories. Among the complexes, **FP1** and **FP3** maintained consistent RMSD values around ~0.18 to 0.21 nm with only minor fluctuations, suggesting stable accommodation within the binding cavity (Table 1). The **FP8** complex showed slightly higher deviation (0.24 to 0.27 nm), though still within an acceptable range, reflecting moderate structural stability of the complex.

**Table 1.** Analysis of MD simulations results for the reference compound, compounds **FP1**, **FP3** and **FP8** and the apo-protein listing different parameters obtained for the ligand-protein complexes in bound form with *Mtb*-DHFR enzyme

No.	Parameters	DprE1				
		Reference	FP1	FP3	FP8	apo form
1	RMSD backbone, nm	0.1979	0.1982	0.1795	0.2295	0.2871
2	RMSD Cα, nm	0.1956	0.1973	0.1799	0.2248	0.1843
3	RMSF, nm	0.1001	0.0872	0.1070	0.1308	0.1094

The RMSF profiles further supported these observations, where most residues fluctuated below 0.20 nm, indicating limited local flexibility. Only a few loop-region residues displayed fluctuations approaching 0.30 to 0.35 nm, which is typical for solvent-exposed or non-structured regions. Importantly, key residues within the binding site, including hydrophobic and aromatic residues such as Trp22 and Phe31, remained conformationally stable throughout the simulation, preserving crucial protein-ligand contacts. Hydrogen-bonding interactions, including water-mediated contacts involving Trp22, persisted for a substantial portion of the simulation timeframe. These interactions, together with stable hydrophobic packing, contributed to the effective retention of **FP1**, **FP3** and **FP8** within the active site, thereby reinforcing their potential as promising inhibitory candidates.

### DFT calculation

Theoretical studies of the synthesized compounds were conducted using the 6-31G** basis set within the Schrödinger suite. This approach facilitated the estimation of essential thermochemical descriptors, namely ionization potential (IP = -*E*_HOMO_), electron affinity (EA = -*E*_LUMO_), electronegativity [*χ* = (IP + EA)/2], chemical potential (*μ* = -*χ*), global hardness [*η* = (IP − EA)/2], softness (*σ* = 1/2*η*), and electrophilicity index [*ω* = *μ*^2^/2*η*]. Collectively, these parameters offer important insights into the molecular reactivity profile. Prior to property evaluation, the designed compounds (**FP1** to **FP9**) were geometrically optimized to obtain *E*_HOMO_ and *E*_LUMO_ values, which are presented in Table 2.

**Table 2.** HOMO, LUMO and the energy gap between the two energy states of the designed compounds (**FP1** to **FP9**)

Compound	HOMO, eV	LUMO, eV	Δ*E* (*E*_LUMO_ - *E*_HOMO_), eV
**FP1**	-0.2249	-0.074	0.1510
**FP2**	-0.2286	-0.079	0.1500
**FP3**	-0.2255	-0.076	0.1493
**FP4**	-0.2262	-0.097	0.1297
**FP5**	-0.2292	-0.101	0.1285
**FP6**	-0.2240	-0.075	0.1489
**FP7**	-0.2281	-0.087	0.1410
**FP8**	-0.2244	-0.075	0.1492
**FP9**	-0.2231	-0.071	0.1521

**Figure 6.**
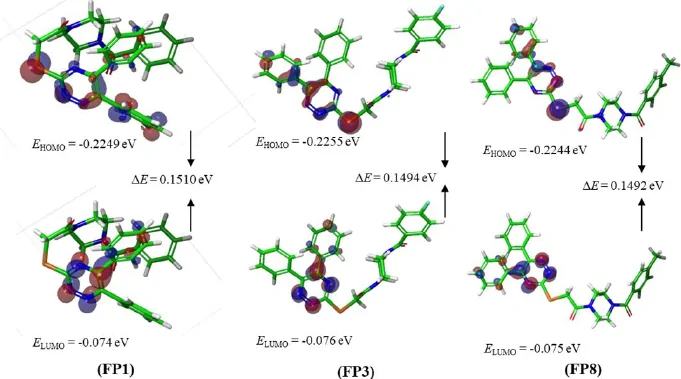
The computed HOMO, LUMO energies and energy gap for compounds **FP1**, **FP3** and **FP8**

The molecular reactivity descriptors calculated for the selected compounds **FP1**, **FP3** and **FP8** (Table 3) indicate closely related but distinguishable electronic characteristics (Figure 6). All three compounds exhibited comparable ionization potential values (0.2249 eV for **FP1**, 0.2255 eV for **FP3** and 0.2244 eV for **FP8**), suggesting a similar tendency to donate electrons. The electron affinity values also fall within a narrow range, reflecting the balanced electron-accepting capabilities of these molecules.

**Table 3.** Molecular parameters of the designed compounds.

Compound	IP, eV	EA, eV	*Χ* / eV	*μ* / eV	*η* / eV	*σ* / eV^-1^	*ω*
**FP1**	0.2249	0.074	0.2618	-0.2618	0.1879	0.0940	0.00644
**FP2**	0.2286	0.079	0.2678	-0.2678	0.1893	0.0946	0.00679
**FP3**	0.2255	0.076	0.2636	-0.2636	0.1874	0.0937	0.00651
**FP4**	0.2262	0.097	0.2745	-0.2745	0.1779	0.0890	0.00670
**FP5**	0.2292	0.101	0.2795	-0.2795	0.1788	0.0894	0.00699
**FP6**	0.2240	0.075	0.2615	-0.2615	0.1865	0.0932	0.00637
**FP7**	0.2281	0.087	0.2716	-0.2716	0.1846	0.0923	0.00681
**FP8**	0.2244	0.075	0.2620	-0.2620	0.1868	0.0934	0.00641
**FP9**	0.2231	0.071	0.2586	-0.2586	0.1876	0.0938	0.00627

In terms of electronegativity, **FP3** (0.2636 eV) and **FP8** (0.2620 eV) show marginally higher values than **FP1** (0.2618 eV), indicating a slightly stronger pull toward electron density. The negative electrostatic potential values further support the stability of the electronic environment of these compounds. Notably, **FP3** exhibits the lowest hardness (0.1874 eV), followed closely by **FP8** (0.1868 eV), indicating greater softness and greater adaptability to electronic redistribution. This behaviour is consistent with their relatively higher softness values, which are often associated with improved chemical reactivity.

Overall, the combined analysis suggests that **FP3** and **FP8** may possess slightly higher reactivity than **FP1**, owing to their lower hardness and favourable softness parameters. However, the proximity of all the calculated values highlights that these compounds share a similar electronic framework, making them promising candidates with balanced stability and reactivity profiles.

### In silico drug likeness and computed pharmacokinetics parameters

All the compounds were evaluated *in silico* for their physicochemical properties (Table 4). Furthermore, the surface area and number of rotatable bonds were found to be within the limits. Each compound displayed varying degrees of water solubility, ranging from moderate to high. Additionally, a range of pharmacokinetic properties was computed using the SwissADME server.

**Table 4.** *In silico* physico-chemical properties of the designed compounds, number of hydrogen bond donors is 0, number of hydrogen bond donors is 1.0459 nm^2^ and rotatable bonds is 8, for all compounds.

Compound	Molecular weight	Number of hydrogen bond acceptors	logP*
**FP1**	495.6	5	3.74
**FP2**	530.04	5	4.1
**FP3**	513.59	6	3.88
**FP4**	574.49	5	4.18
**FP5**	540.59	7	3.01
**FP6**	525.62	6	3.68
**FP7**	530.04	5	4.23
**FP8**	509.62	5	4.02
**FP9**	564.49	5	4.73

*n-octanol/water partition coefficient

### Anti-tuberculosis activity

All synthesized compounds were evaluated for their *in vitro* anti-tubercular activity against *Mycobacterium tuberculosis* H_37_Rv strain, and their efficacy was compared with standard first-line anti-TB drugs, namely isoniazid, ethionamide, and pyrazinamide (Table 5).

**Table 5.** Anti-tubercular activity and cytotoxicity of the synthesized compounds and standard drugs

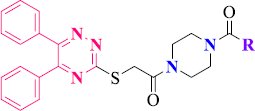			
Compd.	R	MIC, μg mL^-1^ H_37_RV	Cytotoxicity, IC_50_, μg mL^-1^ RAW 264.7^a^
**FP1**	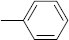	3.12	46.19 ± 0.18
**FP2**	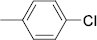	25	ND
**FP3**	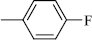	1.6	96.59 ± 0.16
**FP4**	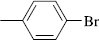	25	ND
**FP5**	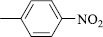	6.2	ND
**FP6**	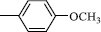	50	ND
**FP7**	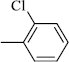	25	ND
**FP8**	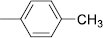	1.6	46.28 ± 0.15
**FP9**	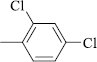	3.12	ND

ND indicates not determined

**^a^**RAW 264.7 (murine macrophage) cell lines. The data was analysed using Graph Pad Prism software

Among the series, compounds **FP3** (4-fluoro) and **FP8** (4-methyl) exhibited the most promising activity, with MIC values of 1.6 μg mL^-1^. This level of activity was found to be comparable to that of isoniazid and ethionamide (MIC = 1.6 μg mL^-1^ for both), suggesting that these compounds possess strong potential as lead molecules for further development. Compounds **FP1** (unsubstituted, -H) and **FP9** (2,4-dichloro) also demonstrated noteworthy inhibition of *M. tuberculosis*, with MIC values of 3.12 μg mL^-1^, nearly equivalent to that of pyrazinamide (MIC = 3.13 μg mL^-1^), a standard second-line anti-TB agent. These results indicate that certain substitutions, such as small electron-donating groups or appropriately placed halogens, can lead to compounds with activity profiles closely resembling those of established drugs. On the other hand, several other derivatives, including **FP2** (4-Cl), **FP4** (4-Br), **FP5** (4-NO₂) and **FP7** (2-Cl), showed moderate to poor activity, with MIC values ranging from 25 to 50 μg mL^-1^, and were significantly less potent than the reference standards. These findings further reinforce the importance of substitution pattern and the electronic nature of functional groups in modulating anti-tubercular activity. Overall, the comparative data suggest that **FP3** and **FP8**, in particular, exhibit a level of efficacy that is on par with first-line anti-TB drugs, making them promising candidates for further pharmacological evaluation.

### Structure-activity relationship for anti-tubercular activity

The structure-activity relationship (SAR) study of the synthesized compounds (**FP1** to **FP9**) against Mycobacterium tuberculosis H37Rv showed that the activity against tuberculosis was significantly affected by the type and size of the substituents on the phenyl ring. For instance, the unsubstituted phenyl derivative **FP1** showed significant activity against the pathogen (MIC = 3.12 μg mL^-1^). This suggests that the phenyl ring itself serves as a privileged structure for the target pathogen. When the phenyl ring was slightly modified with smaller substituents, the activity was further increased, as demonstrated by **FP3**, where the para position on the phenyl ring was occupied by a fluoro group, and FP8, where the para position was occupied by a methyl group, showing the highest activity against the pathogen (MIC = 1.6 μg mL^-1^). In contrast, compounds bearing bulkier halogen substituents, such as chloro (FP2) and bromo (FP4), showed decreased activity, with an MIC of 25 μg mL^-1^. These observations suggest that bulkier substituents negatively affect the compounds’ activity. The presence of a powerful electron-withdrawing group, such as nitro in **FP5**, resulted in moderate activity, with an MIC of 6.2 μg mL^-1^. These observations suggest partial tolerance to electron-withdrawing groups. On the contrary, the presence of a powerful electron-donating group, such as methoxy, in FP6 resulted in decreased activity, with an MIC of 50 μg mL^-1^. These observations suggest negative effects of excessive electron donation. The positional and multiplicity effects were also observed in this series. For example, the monochloro-substituted FP7 showed moderate activity, with an MIC of 25 μg mL^-1^. The dichloro-substituted **FP9** showed increased activity with an MIC of 3.12 μg mL^-1^. These observations suggest the positive effects of increased lipophilicity. The most active compounds in this series showed good cytotoxicity profiles in RAW 264.7 cells.

## Conclusion

In conclusion, the current study aims to report the successful design, synthesis, and extensive biological and computational investigation of a series of phenyl-substituted analogues as prospective anti-tubercular agents. The in vitro screening against M. tuberculosis H_37_Rv has led to the identification of promising inhibitory activities, among which **FP3** and **FP8** were identified as the most potent analogues, possessing low micromolar MIC values. The structure-activity relationship has been established, showing that small hydrophobic substituents on the phenyl ring significantly increase anti-tubercular activity, whereas bulky halogens and highly electron-donating substituents have a detrimental effect on activity. Molecular docking was carried out against both DprE1 and *Mtb*-DHFR, proving that the synthesized compounds have a consistently higher affinity towards *Mtb*-DHFR, surpassing even the reference drug methotrexate, thus identifying DHFR as the most prospective molecular target. The detailed interaction analysis has been carried out, demonstrating that the active compounds bound effectively within the DHFR binding pocket via hydrogen bonding, π-π stacking, and hydrophobic interactions with key catalytic residues. Finally, molecular dynamics simulations were carried out on the most active complexes, demonstrating the structural stability of the ligand-protein complexes, as evidenced by stable RMSD and RMSF profiles and the maintenance of key intermolecular interactions throughout the simulation. Complementary DFT calculations were carried out, providing valuable insight into the electronic properties of the lead compounds, thus proving their highly favourable reactivity profiles, consistent with their biological activities.
